# Outbreaks of blackflies and related problems in Serbia: past and present situation

**DOI:** 10.1186/1756-3305-7-S1-O3

**Published:** 2014-04-01

**Authors:** A Ignjatović Ćupina, D Werner, M Kúdela, L Vujanović, T Brúderová, A Giannelli, M Zgomba, D Petrić

**Affiliations:** 1 Faculty of Agriculture, University of Novi Sad, Novi Sad, Serbia; 2 Leibniz Centre for Agricultural Landscape Research (ZALF), Müncheberg, Germany; 3 Faculty of Natural Sciences, Comenius University, Bratislava, Slovakia; 4 Department for Dermatology and Venereology, Clinical Center of Vojvodina, Novi Sad, Serbia; 5 Department of Veterinary Medicine, University of Bari, Valenzano (Bari), Italy

## 

Due to the repeated outbreaks of blackflies, consequent economical losses and health problems, Serbia was considered as the most threatened European country in the past. During the last century (up to `60s), *Simulium colombaschense *caused enormous losses of livestock. Significant losses in poultry production caused by *S. maculatum *were also reported in 1958, while *S. erythrocephalum *caused severe dermatological problems in humans in 1965 and 1970.

In the last fifteen years, repeated outbreaks of blackflies and reemerging of bite related problems in humans were recorded in some parts of Serbia.

The research objective was to update the knowledge of blackfly pest species distribution in Serbia, with a special attention to endangered regions in the present and the past. Samplings were conducted in the period 2003-2012. Immature stages were collected from submerged substrates: in the Danube river and its tributaries, the Nera river and the Nišava river. Adults were sampled close to the breeding sites by application of CO_2 _baited traps or by light traps.

In the lowlands 11 blackfly species were recorded. Two mammophilic species have been dominant: *S. erythrocephalum *in the Danube and *S. ornatum *(complex) in confluent streams. In 2010, *S. erythrocephalum *was recorded for the first time in the hilly area in southeastern Serbia. Periods of high adult population density of those two species coincided with the bite cases reports.

In the Iron Gate region, 21 mainly mammophilic species were recorded in the Danube confluents exclusively. *S*. *colombaschense*, the main pest species in the past, was detected only in the Nera river, about 30 km upstream from the entrance of the Iron Gate.

Present state of blackfly fauna composition indicates the existing risk of outbreaks of some species in the future.

Acknowledgements: The study was supported by The Ministry of Education, Science and Technological Development of the Republic of Serbia (projects TR31084 and III43007).

